# User adaptation in Myoelectric Man-Machine Interfaces

**DOI:** 10.1038/s41598-017-04255-x

**Published:** 2017-06-30

**Authors:** Janne M. Hahne, Marko Markovic, Dario Farina

**Affiliations:** 1 Neurorehabilitaiton Systems Research Group, Department of Trauma Surgery, Orthopedic Surgery and Hand Surgery, Universiy Medical Center Göttingen, Göttingen, Germany; 2 Department of Bioengineering, Imperial College London, London, UK

## Abstract

State of the art clinical hand prostheses are controlled in a simple and limited way that allows the activation of one function at a time. More advanced laboratory approaches, based on machine learning, offer a significant increase in functionality, but their clinical impact is limited, mainly due to lack of reliability. In this study, we analyse two conceptually different machine learning approaches, focusing on their robustness and performance in a closed loop application. A classification (finite number of classes) and a regression (continuous mapping) based projection of EMG into external commands were applied while artificially introducing non-stationarities in the EMG signals. When tested on ten able-bodied individuals and one transradial amputee, the two methods were similarly influenced by non-stationarities when tested offline. However, in online tests, where the user could adapt his muscle activation patterns to the changed conditions, the regression-based approach was significantly less influenced by the changes in signal features than the classification approach. This observation demonstrates, on the one hand, the importance of online tests with users in the loop for assessing the performance of myocontrol approaches. On the other hand, it also demonstrates that regression allows for a better user correction of control commands than classification.

## Introduction

Losing a hand is a highly traumatic incident that imposes a radical change of life. Simple activities of daily living, such as eating, dressing and body care, have to be relearned completely and in many cases, the learned occupation cannot be continued. The regions in the central and peripheral nervous system that used to control the lost limb usually remain intact after an amputation and the person typically experiences a phantom limb^1^, perceived as the natural limb. When executing motions with the phantom limb, the remnant muscles above the amputation contract and generate electromyographic (EMG) signals.

Electrically powered hand prostheses controlled by EMG signals of the residual muscles have been commercially available for decades^2^. In the conventional control scheme that is used by all manufactures of myoelectric hand prostheses, two active electrode systems are integrated into the prosthetic socket above a pair of residual antagonist muscles (e.g., flexor and extensor in the forearm or biceps and triceps in the upper arm). For example, in transradial amputations, extension of the phantom wrist is usually mapped into opening of the prosthesis and flexion into closing^3^. The speed of the prosthesis actions is often controlled by varying the intensity of contraction (proportional control). With this simple approach, it is possible to activate one degree of freedom (DOF) at a time. Extending the approach by recording from additional electrode pairs, placed on antagonistic muscles, is not possible, as usually there are not sufficient independent control sites. Therefore, the two-channel scheme is typically extended to more DOFs by maintaining only two control signals and switching the control to a second DOF through the switch function. The switch is practically controlled by a trigger generated by the user (e.g. co-contraction) or similar heuristics. This type of control is slow and unnatural, and contrasts with the rich potential of modern robotic limbs^4^.

To overcome the limitations of conventional myocontrol, significant research effort has been invested in machine learning based approaches^5–10^. State of the art methods are based on time and frequency domain features^11^, extracted from typically 4 to 10 EMG signals and associated to prosthesis motions by a classifier. The classification approach is also often combined with force estimation, based on EMG amplitude, to allow for proportional control of each prosthetic action^12^. Moreover, combined motions can be identified by introducing additional classes^13^.

More recently, regression based myoelectric control has been introduced^14–21^ Contrary to a classifier, a regressor provides a continuous output for each DOF independently. This allows for a free combination of all estimated DOFs, which has been termed as simultaneous and proportional control of multiple DOFs^19, 22^. As the activation level is estimated for each function independently, the user can e.g. perform a slow wrist rotation while opening the hand with high speed in multi-DOF prostheses. It has been often claimed that regression is superior to classification for myocontrol^22^, although the two approaches have not been compared so far.

It is well recognized that the critical aspect to test when comparing myocontrol algorithms for real-life applications is the robustness with respect to factors changing over time^22–25^. Factors that have been reported to negatively influence the performance of myocontrol over time are electrode misplacements, changes in the electrode-skin interface due to e.g. sweating^22^, changes in arm-position^26^, and time between the training and testing^27, 28^. However, studies assessing the impact of these factors have been usually performed in offline conditions, without the possibility for the user to adapt to the changed conditions. Nonetheless, user adaptation may substantially influence online performance with respect to results obtained offline^29^. When the user controls the system online and receives continuous feedback on the generated commands, he/she can indeed learn to compensate for the changed signal features and therefore counteract the non-stationarities. Although not yet demonstrated, different types of EMG mapping would presumably allow different degrees of compensation by the user.

In this study, we hypothesized that, when applied online, a regression based approach allows for better user compensation of non-stationarities with respect to a classification-based myocontrol. *We expected* that the two approaches would significantly differ in performance when tested against non-stationary signal features during online, but not offline, control. We therefore investigated the influence of artificially generated signal distortion on linear discriminant analysis (LDA) (classification) and linear regression (LR), both offline and with the user in the loop (online).

## Methods

### Subjects

Ten able-bodied subjects (23–34 years, eight males and two females, all right handed) and one subject with trans-radial amputation (left side, 56 years, 36 years after amputation) participated in this study. The experiments were in accordance with the declaration of Helsinki, the local ethic committee approved the experiment (Ethikkommission der Universitätsmedizin Göttingen, approval number 22/04/16) and all participants gave their informed consent.

### Experimental Setup

**Figure 1:**
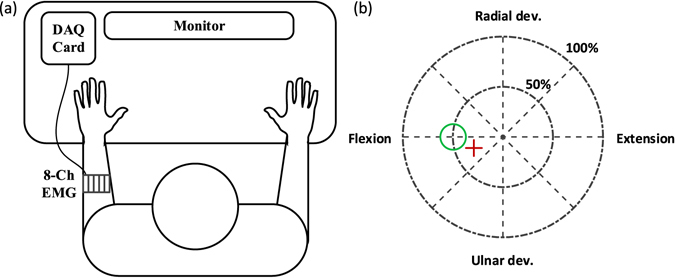
(**a**) Experimental setup, consisting of eight Otto Bock 13E200AC EMG electrodes equally spaced around the forearm and powered by a prosthesis battery pack, a National Instruments Data Acquisition (DAQ) card, and a PC with separate user screen. (**b**) Feedback window in real-time control tasks. The user controls the position of the red cross with his/her EMG signals, utilizing either a classifier or a regressor. The task is to move and keep the red cross into the green circle in the conditions tested.

The experimental setup is shown in Fig. 1(a). During the experiments, the subjects seated in a chair in front of a computer screen. The arm was placed on a soft foam pad on the arm-rests of the chair in a comfortable position with the elbow slightly flexed and the thumb pointing upwards. Eight dry, active Otto Bock EMG electrodes (13E200AC) were placed around the dominant forearm of the able-bodied subjects and the forearm of the side of amputation of the subject with amputation. The skin was moisturized with water and a custom-designed spring system ensured a constant pressure and equal spacing of the electrodes around the forearm (Fig. 1(a)). The electrodes were powered by a conventional 7.2 V Li-Ion prosthesis battery; they provided amplified and filtered EMG signals that were recorded with a National Instrument USB-data-acquisition device 6251. The sampling frequency was 2000 Hz.

### Signal Processing

The signal processing and display were performed in MATLAB, running on an i5-2500, 3.3 Ghz, personal computer with 8 GB RAM. The data were processed in blocks of 40 ms that corresponded to the update rate of the entire system, including the real-time display.

To reduce movement artifacts and maximize the signal-to-noise-ratio, the data were digitally filtered by a 4th order Butterworth band-pass filter with a pass-band between 30 and 500 Hz. An additional 50 Hz comb filter was applied to remove power-line interferences, including its harmonics. After pre-processing, the data were transferred into a queue buffer so that the last 4 s of data were available for feature extraction and real-time display. In parallel, the raw, unfiltered signals were stored on a hard-disk so that they were available for training of the machine learning algorithms and offline analysis.

The root mean square (RMS) was computed as feature for each EMG channel from intervals of 160 ms. Due to the increment of 40ms, adjacent blocks were overlapping by 120 ms. This window duration is within the acceptable time delay between user command and prosthesis reaction^30^. The same feature was used for both the regression and the classification based approaches.

### Regression

Regression consisted of a linear mapping W between the features φ and the two dimensional output y:

1 $$\hat{y}={W}^{T}{\rm{\phi }}$$

The regression coefficients W were obtained by ordinary linear regression (LR), i.e. by minimizing the mean quadratic error for the three calibration runs^31^. The solution is given in a closed form as:

2 $$W={({{\rm{\Phi }}{\rm{\Phi }}}^{T})}^{-1}{\rm{\Phi }}Y$$

where Φ and *Y* are matrices containing all training samples *φ*(*t*) and *y*(*t*) as column vectors.

Due to the stochastic nature of the EMG, mapping the estimate $\hat{y}(t)$ directly into the position of a cursor would give a relatively unstable control. Therefore, a soothing filter was applied as post processing, as commonly done in regression based myoelectric control^32^. We selected an exponential moving average filter, with a filter constant of 1/25, which has the advantage of a relatively fast reaction time with respect to alternative filters without introducing an overshoot^33, 34^.

### Classification

For the classification-based approach, we applied a multiclass linear discriminant analysis after Fisher (LDA). The LDA classifier decides for that class $c\in \{1\ldots C\}$ with the largest posterior probability.

3 $$\hat{y}={\rm{\arg }}\,\mathop{{\rm{\max }}}\limits_{c}\,{\delta }_{c}(\phi )$$

where the linear discriminant function *δ*_*c*_(*φ*) is given by

4 $${\delta }_{c}(\phi )={\phi }^{T}{\sum }^{-1}{\mu }_{c}-\frac{1}{2}{{\mu }_{c}}^{T}{\sum }^{-1}{\mu }_{c}+\,\mathrm{log}\,{\pi }_{c}$$

with Σ being the pooled covariance matrix of all samples in feature-space, *μ*_*c*_ the class-specific mean and *π*_*c*_ = 1/*C* the prior probability, here assumed to be equal for each class.

The LDA classification model was trained using individual classes for single and combined motions and the rest class, i.e. in total nine classes were used. For the eight non-rest classes, both the dynamic and the static parts of the movements were used. To increase the stability, a majority vote was applied to the last 5 classifier decisions^35^.

As the classifier estimates only the type of movement (direction of the wrist) but not the level of activation, the contraction strength was estimated by the average RMS across all channels. Since the average RMS varies across classes, class specific scaling factors were applied that were determined from the static parts of the calibration runs. To stabilize the output and for comparison with regression, an EMA smoothing filter with the same filter constant as in the regression case was applied on the contraction level estimation.

In this way, both the regressor and the classifier allowed for a simultaneous and proportional control of two DOF.

### Experiments

The experiment involved real-time myoelectric control tasks with a cursor on a computer screen; the task was executed either with the regressor, or with the classifier under the influence of artificially added signal degradation. This was obtained by adding white noise to the recordings, either progressively or suddenly. The able-bodied subjects performed non-restricted movements of the two wrist DOFs (i.e., flexion/extension and radial/ulnar deviation), including combined motions to generate the EMG signals. Similarly, the subject with amputation performed 2-DoF movements with his phantom limb but excluding combined motions, as he was not able to conduct them in a reliable manner without appropriate feedback. The tests consisted in two sessions on two consecutive days. In each session, the subjects performed the online control task with either classification or regression of the EMG. The order of the sessions was randomized in a balanced way, so that half of the subjects performed the regression control in the first day and the other half in the second day. An overview of the experimental protocol is provided in Table 1.

**Table 1:** Overview on one session of the experimental protocol.

Phase	Number of runs	Type	Artificial noise
1	3	Calibration	—
2	X	real-time validation	—
3	6	real-time without noise (open-loop analysis)	—
4	16	linearly increasing noise (closed-loop)	0.1 × *A*_max_ × *t*
5	16	sudden noise (closed-loop)	0.4 × *A*_max_

Annotations: X – the tests were performed until satisfactory performance was reached (see text for explanation); *A*_max_ – maximal EMG amplitude in the calibration phase during flexion/extension movements; t – time.

First, the signal quality was checked by visual inspection of the raw EMG signals during rest and contractions. The participant was trained in performing the motions while receiving a real-time display of the amplitudes of the eight EMG signals. This was particularly needed for the subject with amputation, who did not have the intrinsic feedback of the actual limb to optimize the phantom limb movements. Then the subjects conducted three calibration runs, in which they performed contractions by following a cursor that moved along fixed, pre-defined trajectories within a two dimensional coordinate system (cue-based trajectories). In this coordinate system the origin corresponded to rest, the horizontal axis to the DOF flexion/extension, the vertical axis to radial/ulnar deviation, and the unity circle to a maximal voluntary contraction (MVC) (Fig. 1(b)). The cue-based trajectories corresponded to the cursor moving at constant speed from the origin to 80% of MVC in 2 s, remaining in the target position for 4 s, and returning to the origin in 2 s. Eight directions were defined at increments of 45 degrees, corresponding to four single-DOF motions and four combined motions. Additionally, 8 s of rest were recorded within each calibration run.

The machine learning method (either the regressor or the classifier) was trained based on the three calibration runs. Then a real-time control was activated in which the EMG signals were mapped by the machine learner into the position of the cursor (position control). According to this control scheme, the distance of the cursor from the origin increased with increasing contraction intensity. While the regressor spanned continuously the 2D control space, the classifier restricted the cursor position to eight discrete and equispaced axes. The classifier included a proportional control for each direction so that the distance from the origin could be controlled continuously with both control methods. With this position control scheme, the user receives a direct feedback on the estimate of the machine learning algorithm. For classification, the position of the cursor, which is restricted to the eight axes, gives a direct and intuitive feedback on the estimated class. In contrast to velocity control, during the position control, the regions beyond the four main axes can only be reached by performing a combined motion and not by a sequential control of two non-combined motions.

After a familiarization of 5–10 min with the online control, the user had the task to hit a series of circular targets with the cursor (phase 2). The targets appeared randomly at the eight directions and at a distance to the origin corresponding to 50% MVC. Before each motion-target, a “rest-target” appeared at the origin to ensure that the user relaxed before each reaching task. The radius of the target circles was 0.15 units. The reaching task was considered successful when the user could hit the target in less than 10 s and dwelled there for 1 s. These runs were repeated until the subject felt comfortable with the control and was able to enter all targets at least once, and to reach the dwell time of 1 s for at least 5 of 8 targets. The aim of this phase was to achieve a level of good overall control performance with both control approaches.

In phase 3, each subject was asked to hit three targets for the flexion and three for the extension direction without any added disturbance. In contrast to phase 2, the targets did not disappear after a certain dwell time but remained at the same position for the full 20 s in each run. These data were used for the offline (open-loop) analysis by adding noise to the signals and repeating the analysis offline (Table 1).

In phase 4 and 5, the task was similar as in phase 3, but artificial noise was added during the online control (closed-loop). The subjects were informed about the added noise and were asked to try to compensate for the artificial disturbance. They performed 16 reaching runs in each phase (2 movement directions with noise on each of the 8 EMG channels) (Table 1). Noise was added with amplitude defined as percentage of the maximum average amplitude (across all channels) *A*_max_ of the three calibration runs for flexion and extension.

The noise was added either progressively (phase 4) or suddenly (phase 5). For the progressive disturbance, the white Gaussian noise was added to one of the eight raw EMG signals after 5 s from the beginning of the run, with a linearly increasing amplitude (RMS) according to 0.1 × *A*_max_ × *t* until the total duration of 20 s. The sudden disturbance corresponded to a step-wise summation of noise after 5 s from the beginning of the run, with an intensity of 0.4 × *A*_max_ for 10 s. Thus, the runs in phase 5 had a total duration of only 15 s. The reason for including only two target positions in phase 3–5 was to keep the duration of the experiment and the subject’s fatigue within reasonable limits. Additionally, our preliminary tests have demonstrated that varying the electrode channel at which the noise was introduced and not the target size/position had the decisive effect on the subject’s performance.

The main focus was then to compare the deviation of the two methods from their baseline when introducing the non-stationary conditions in the signal features. It has to be noted that, despite a similar familiarization with the two methods and the achievement of controllability, as defined above, by all subjects, it was not possible to reach the same quantitative performance for the baselines of the two methods (see results). For this reason, performance metrics were computed in relation to the baseline for each subject and method (see below), i.e. the focus on the comparison was entirely on robustness and not on absolute performance.

### Offline Analysis

To investigate the impact of artificial noise on both machine learning methods, offline analyses (open loop control) were conducted with the data collected in phase 3 (Table 1). The noise was added in the same way as in the closed-loop tests of phases 4 and 5. In this way, the impact of the noise on the algorithm could be investigated separately from the potential compensation due to the user (i.e., closed loop vs. open loop control).

### Performance Metrics and Statistical Analyses

Four previously defined metrics^36, 37^ were used to validate the real-time performance with targets located in all eight directions (phase 2). These were the completion rate, completion time, overshoot ratio, and path efficiency. The completion rate is the average ratio between successfully hit targets and total number of targets, completion time the average time needed to hit all successful targets, overshoot ratio the number of times a target was left before the 1-s dwell time, normalized by the total number of targets, and path efficiency the average ratio of the most efficient and actually traveled path-length.

The performance metrics (user-error) for the offline and online tests under the influence of artificial noise (phase 3–5) was the Euclidian distance between the centre of the target and the cursor over time. Additionally, the mean error in the time interval 4–5 s, i.e. immediately before adding the artificial noise was measured as a baseline error without noise. Since the system was calibrated relative to the user’s MVC, the feedback window and thus the overall task was expressed relatively to it. More specifically, the radius of the outer feedback circle (Fig. 1(b)) was of unit length and corresponded to 100% MVC. In the following, we will refer to units of error when discussing the performance metrics in online and offline analysis.

The focus of this study was assessing the deterioration of the control with respect to the baseline. Therefore the decrease in performance due to the artificial disturbance was expressed as an increase in the baseline error. Consequently, when calculating the resulting error the offset baseline performance was removed by subtraction, leaving only the increase in error due to the non-stationarity (error determined by the artificial noise). As measure of robustness, in the linearly increasing noise experiments, we quantified the time interval during which the user could keep the residual noise error below 0.3 units above the baseline error, despite the increase in noise level. In the sudden noise experiments, the residual error after baseline error subtraction was measured in the interval 14–15 s.

Since the performance indexes were not always normally distributed (Lilliefors test), the non-parametric Wilcoxon signed-rank test was used for the comparisons between LDA vs. LR or online vs. offline, respectively. The significance level was set at p = 0.05. When more than two conditions were tested, a Bonferroni correction for pairwise comparisons was performed.

## Results

### Overall Controllability

**Figure 2:**
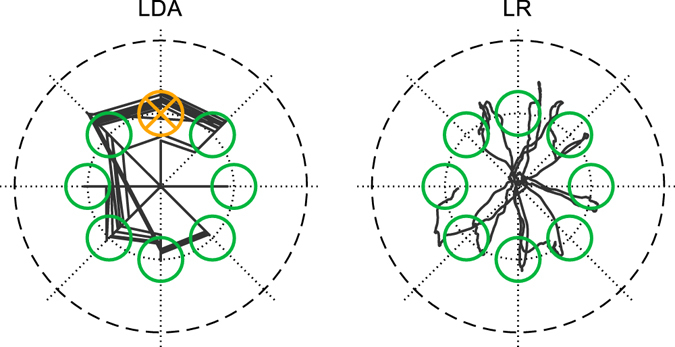
Qualitative presentation of one real-time validation run for the regression-based and for the classification-based control for a representative subject. The black curve indicates the trace of the curser and the circles the target positons. Green circles were successfully hit; yellow circles were entered at least once but not hit due to insufficient dwell time. LDA: Classification by linear discriminant analysis; LR: Linear regression.

Figure 2 shows a qualitative representation of the online validation runs (phase 2) for both methods for a representative subject. The corresponding quantitative results are provided below. In both methods the majority of the targets were reached successfully. For LDA, the movement space was limited to eight directions. In this case, some targets were reached directly with the right direction, while for other targets, the wrong class was sometimes temporarily selected, as indicated by the straight trace-lines between the eight axes of Fig. 2. For LR, the full 2D movement space could be covered.

In the validation runs, the completion time (LDA: 3.79 ± 1.49 s, LR: 3.56 ± 0.87 s) was not different for the two methods. On the other hand, the completion rate (LDA: 0.72 ± 0.14, LR: 0.99 ± 0.04, P < 0.001), overshoot ratio (LDA: 2.18 ± 2.82, LR: 0.14 ± 0.14, P < 0.001) and path efficiency (LDA: 0.27 ± 0.12, LR: 0.56 ± 0.09, P < 0.001) were significantly different between LDA and LR. There were no significant differences in performance between non-combined and combined targets.

### Offline Analysis (Open Loop)

**Figure 3:**
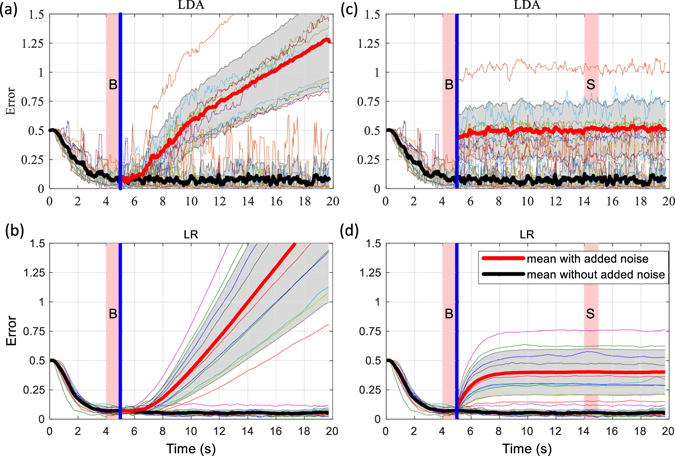
Open loop control performance (offline analysis). (**a**,**b**) Error in reaching when linearly increasing noise is added to the signals offline for LDA (**a**) and LR (**b**). (**c**,**d**) Error in reaching when sudden noise was added to the signals offline for LDA (**c**) and LR (**d**). The plots show the error for each subject, the mean (without noise: black lines, with noise: red lines) and standard deviation (grey area is ±SD) across all subjects. The blue line indicates the beginning of the interval when the artificial noise was added and the pink shaded areas labelled with “B” and “S” mark the periods when the baseline error and the error with noise were extracted.

Figure 3 shows the results of the offline analyses. It displays, for each subject, the distance of the cursor to the centre of the target over time, averaged across the six runs (three repetitions for each of the two movements) and all noise cases. Without artificial noise, the average baseline error was 0.08 ± 0.06 units for LDA and 0.07 ± 0.03 units for LR (not significantly different, P > 0.05). The error was then evaluated for the interval with noise and, for proper comparison, expressed as residual error after baseline subtraction. In this way, the comparison focused only on the robustness, i.e. the increase in error due to the added noise.

For the linearly increasing noise (Fig. 3(a,b)), the error increased with the noise level for both methods. The average time when the error exceeded the selected threshold of 0.3 units above the subject specific baseline error was not significantly different between the two methods (p > 0.05; LDA: 3.10 ± 1.80 s; LR: 5.22 ± 1.70 s).

For the sudden noise (Fig. 3(c,d)), the error increased significantly with respect to the baseline by a similar amount for the two methods (not statistically different, p > 0.05, LDA: 0.43 ± 0.22 units; LR: 0.33 ± 0.18 units).

In summary, the impact of the noise in the offline tests, when expressed as an increase in the baseline error of each condition, was the same for both methods. Therefore, any difference observed in the error of the online analysis should be due to a different capacity of adaption by the user when included in the loop.

### Online Tests (Closed Loop)

**Figure 4:**
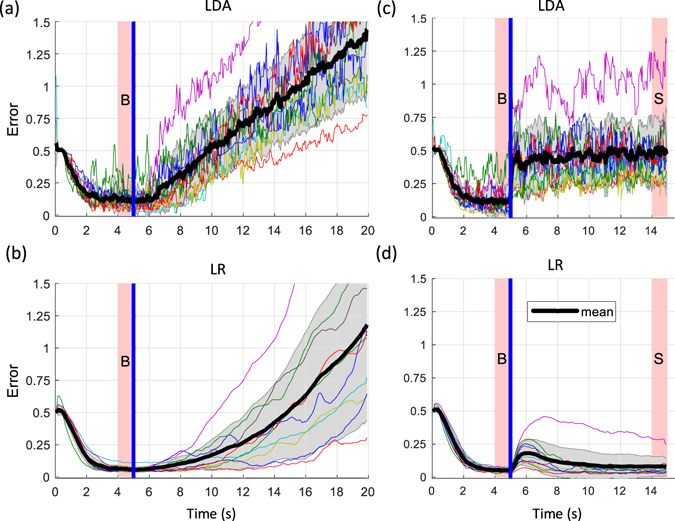
Closed loop control performance (online). (**a**,**b**) Error in reaching when linearly increasing noise was added to the signals online for LDA (**a**) and LR (**b**). (**c**,**d**) Error in reaching when sudden noise was added to the signals online for LDA (**c**) and LR (**d**). The plots show the error for each subject and the mean and standard deviation across all subjects, for LDA (**a**) and LR (**b**). The plots show the error for each subject, the mean (black lines) and standard deviation (grey area is ±SD) across all subjects. The blue line indicates the beginning of the interval when the artificial noise was added and the pink shaded areas labelled with “B” and “S” mark the periods when the baseline error and the error with noise were extracted.

Figure 4 shows the results of the online tests. For the linearly increasing noise (Fig. 4(a,b)), the time interval for which the subjects could keep the error below the threshold of 0.3 units above baseline error was significantly smaller for LDA (3.47 ± 1.68 s) than for LR (9.53 ± 3.29 s) (P < 0.001).

For the sudden noise experiments (Fig. 4(c,d)), LDA showed a similar reaction in the closed-loop scenario as in the offline simulation. The error with noise (0.49 ± 0.24 units) was significantly larger than the baseline (0.12 ± 0.10 units) (P < 0.001). For LR, the closed-loop tests with sudden noise showed different results than the corresponding offline simulations. The error increased from a baseline of 0.05 ± 0.01 units and reached a peak of 0.19 ± 0.12 units after 1.04 ± 0.49 s. Then, the user was able to compensate for the disturbance and decrease the error back to 0.08 ± 0.07 units, which was not significantly higher than the baseline.

### Comparison Open Loop – Closed Loop

**Figure 5:**
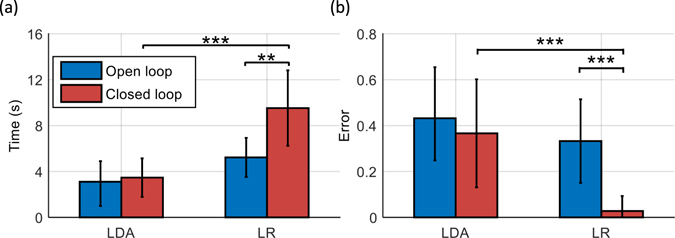
Duration, for that each subject was able to keep the error in the linearly increasing noise scenario below 0.3 units above his/her individual baseline error (**a**). Baseline error increase in the sudden noise scenario (**b**). The plots and error-bars show inter-subject means and standard deviations. The differences between LDA vs. LR and open loop vs. closed loop were tested on statistical significance by Wilcoxon rank sum tests with Bonferroni correction and significant differences are marked with stars (*P < 0.05; **P < 0.01; ***P < 0.001).

The time during which the error remained below the threshold of 0.3 units above the individual baseline error in the linearly increasing noise experiments is summarized in Fig. 5(a) for the offline and online analyses. Similarly, the error increase in the sudden noise experiments is summarized in Fig. 5(b). The differences between LDA vs. LR and open loop vs. closed loop were tested on statistical significance by Wilcoxon rank sum tests with Bonferroni correction.

In the linearly increasing noise scenario, LDA showed no significant differences when comparing the open loop with the closed loop case. Conversely, with LR the user was able to compensate significantly longer for the constantly increasing noise in the closed loop case compared to the open loop case (P < 0.01) (Fig. 5(a)).

Also for the sudden noise, the performance of LDA did not significantly change from the open loop to the closed loop case. Conversely, LR showed a significant improvement in the closed loop case compared to the open loop case (P < 0.001) (Fig. 5(b)).

In summary, the user adapted and significantly improved the performance in the closed loop case with respect to the open loop case with LR but not with LDA.

Finally, we also repeated the same tests as above on three able-bodied subjects but with only four classes plus rest for the LDA and the full time-domain feature-set^11^ for both LDA and LR. Details of these results are not presented. Briefly, in this case, the baseline performance of LDA increased and was comparable to LR. Nevertheless, all other tests with noise (offline/online) led qualitatively to the same results as described above for the main experimental session. Therefore, we exclude that the difference in baseline performance between LDA and LR in the able-bodied subjects for the main experimental session determined the different online behaviour.

### Transradial amputee

The subject with transradial amputation performed similar tasks as the able-bodied subjects. However, initial tests that included the combined calibration motions led to very poor results, as he was not able to reliably generate combined contractions in the calibration procedure. Therefore, combined classes were removed from the training protocol for this subject and the LDA was reduced to four motion classes and the rest class. All tests were therefore done with four target positions and lasted 15 s. Beside this change, the tests were the same as for the able-bodied subjects.

**Figure 6:**
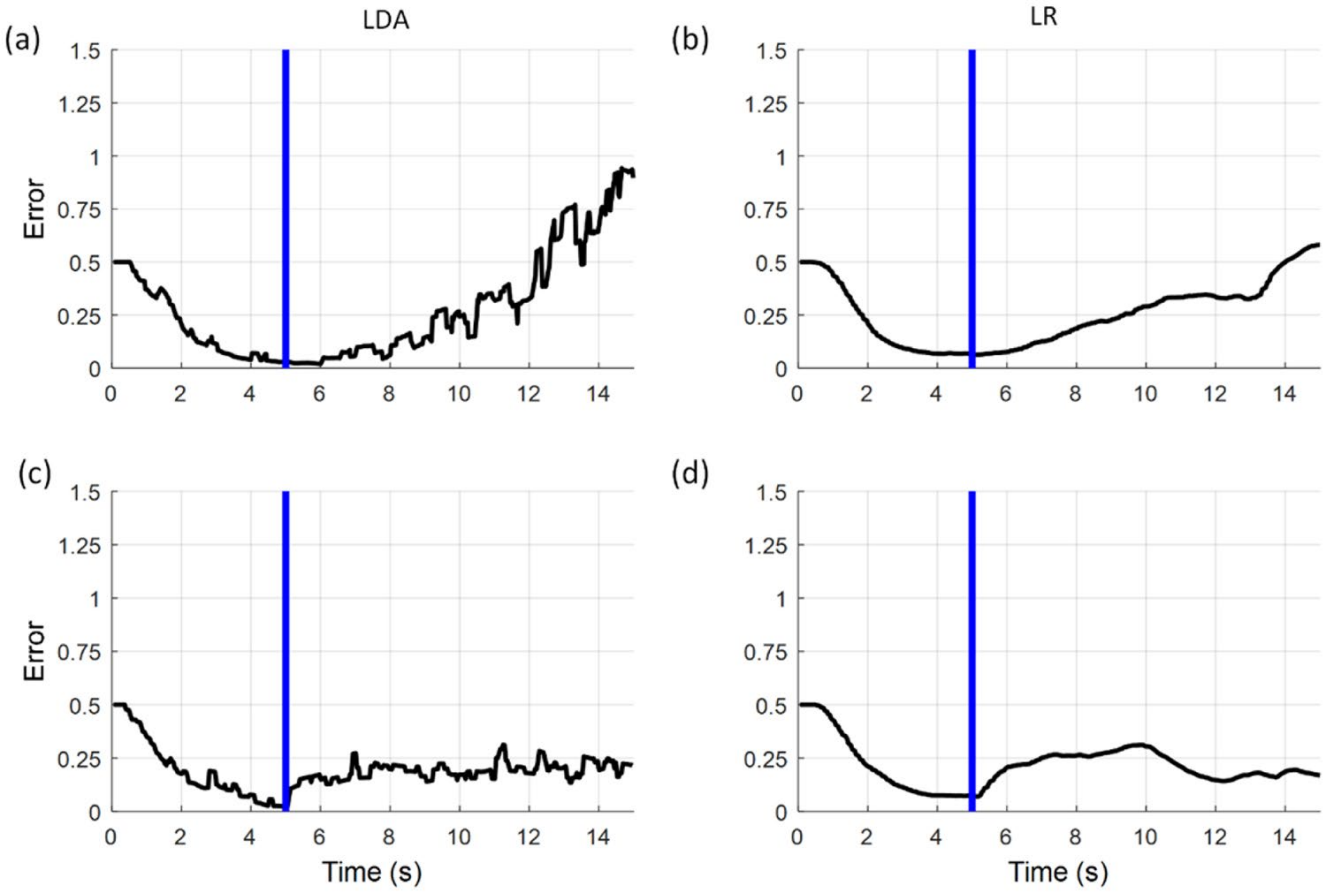
Results of closed-loop experiments for subject with transradial amputation. (**a**,**b**) Error in reaching when linearly increasing noise was added to the signals online for LDA (**a**) and LR (**b**). (**c**, **d**) Error in reaching when sudden noise was added to the signals online for LDA (**c**) and LR (**d**). All four scenarios show similar trends as the closed-loop experiments with able-bodied subjects.

The baseline error was 0.04 units for LDA and 0.07 units for LR. With respect to able-bodied subjects, this value was smaller for LDA and slightly greater for LR. For the linearly increasing noise scenario (Fig. 6(a,b)), the subject could maintain the error below threshold for 5.46 s with LDA and 8.25 s with LR, in accordance with the able-bodied subjects.

As for the other conditions, the amputee subject behaved similarly as the able-bodied subjects when faced with the step-noise (Fig. 6(c,d)). The error he produced increased when noise was added and he was able to partly compensate for it with LR but less so with LDA. The error level at the intervals 4–5 s and 14–15 s were 0.04 and 0.21 for LDA and 0.07 and 0.18 for LR.

## Discussion and Conclusion

We have investigated the potential of user adaptation to non-stationary EMG signal features when using two advanced schemes of myocontrol (regression and classification). The non-stationarities were simulated by adding white noise to one of the EMG channels, so that they could be controlled and repeated in all conditions, while the subjects performed reaching tasks. The results are the first to directly compare classification with regression based control and their robustness to non-stationarities in a closed-loop setup, where the user can adapt and potentially compensate for the external disturbance.

The offline analysis demonstrated that both LDA and LR are similarly affected by artificially added noise when only the algorithm running in an open loop is considered. However, when the user is integrated into the loop (i.e., the loop is closed, as in the real-time scenario) there were significant differences in performance when using LDA and LR. In Regression, the user could partly compensate for the added noise whereas classification did not result in significant differences between online and offline. This indicates that a correction by user adaptation was possible for LR but not for LDA.

Two factors may influence the user’s overall ability of correcting input signal non-stationarities with LDA and LR. First, with classification, the mapping is continuous only in amplitude (proportional control) but not in direction (discrete number of classes). In case of misclassification, the user would therefore receive a feedback whether the corrective attempts are beneficial only when a class-boundary is crossed. With regression, the mapping is continuous in both direction and amplitude. Therefore, the user obtains a direct feedback whenever he/she adapts the muscle contractions and can thus better find an optimal corrective muscle activation pattern. This is also related to the clinical observation that some patients cannot improve their performance in classification even after a long training and require a continuous feedback on EMG signals to be able to reach a good performance^38^. Second, there may be a difference between algorithms in terms of the available solution space. For example, for certain non-stationarities as artificially induced in the current study, there may exist a corrective muscle activation pattern within the space of physiological possible contractions for LR but not for LDA.

The differences between the offline (open-loop) and the online (closed-loop) performance demonstrate that offline investigations are not sufficient for testing myocontrol schemes and emphasizes the need to include the user into the loop, possibly in tasks that include non-stationary conditions.

Even if we targeted a similar baseline performance for both methods and trained the subjects until a certain performance was reached, LDA with combined movements led to a slightly greater baseline error for the able-bodied subjects. It is possible that more advanced approaches to extend the classification to combined motions^39^, may reduce the reference baseline error for LDA. However, our main aim was to study the degradation of control with noise and, when we compensated for the baseline error, the behaviour in offline analyses was comparable for the two approaches tested. *Although additional time*-*domain features are often used for LDA*, *we decided to use the RMS features in both LR and LDA*. *This allowed for a fair comparison as the artificially added noise may impact different features differently*. *In addition*, *the systems were tested with eight classes and the rest class in order to provide simultaneous control in both methods*. *As we are aware that these design decisions may influence the outcome of the study*, *we conducted an additional pilot study on three able*-*bodied subjects* (*not shown*) *where we utilized the time*-*domain feature*-*set* ^11^ *for both LR and LDA and reduced the number of classes in LDA to five* (*four non*-*combined and rest*). *In these additional tests*, *the LDA baseline performance* (*offline and online without noise*) *improved*, *as expected*, yet the relative error in both offline and online tests replicated the one of the main experimental analysis reported for nine classed and RMS feature.

However, the reduction to non-combined motions implies a significant reduction in functionality. When only non-combined movements are used, complex tasks have to be split into several sub-tasks that have to be executed sequentially, similar as in conventional, commercially available prostheses. In the view of advanced control functionality, we focused on a rather large number of classes (allowing for simultaneous motions) but we compared both control methods in the same conditions (two motions). Finally, it has to be noted that LR has the additional advantage with respect to classification that combined motions can even be estimated when trained with non-combined motions only 19.

This study investigated the real-time compensation capabilities to non-stationarities by the user when two conceptually different control techniques were used with artificially added noise. To avoid fatigue and loss of motivation, we limited the duration of the experiments, so that we tested the real-time adaptation on two motions and one contraction-level only. Other factors could be varied in future work, such as the adaptation capabilities for different target distances or potential differences between non-combined and combined motions.

In conclusion, despite similar offline performance with added noise, regression and classification myoelectric interfaces were significantly different when analysed for their robustness to non-stationary signal features during online control. Regression provided a continuous mapping in the position space and allowed for compensation of the added noise by the user. Conversely, the user did not adapt against the added noise with classification. This result is the first that demonstrates the superiority of recently proposed regression schemes for myocontrol over more established classification approaches when tested for non-stationary conditions. It also highlights that influencing factors identified as detrimental for myocontrol may have a more limited effect for a myoelectric regression interface than previously identified for classification.
